# Prognostic value of the preoperative lymphocyte-to-monocyte ratio for survival after lung cancer surgery

**DOI:** 10.1186/s12890-021-01446-1

**Published:** 2021-03-02

**Authors:** Ricard Ramos, Ivan Macía, Arturo Navarro-Martin, Carlos Déniz, Francisco Rivas, Anna Ureña, Cristina Masuet-Aumatell, Camilo Moreno, Ernest Nadal, Ignacio Escobar

**Affiliations:** 1 Department of Thoracic Surgery, Hospital Universitari de Bellvitge, Bellvitge Biomedical Research Institute (IDIBELL), Feixa Llarga s/n., 08907 L´Hospitalet de Llobregat, Barcelona, Spain; 2 Department of Radiation Oncology, Catalan Institute of Oncology (ICO), Bellvitge Biomedical Research Institute (IDIBELL), L’Hospitalet de Llobregat, Barcelona, Spain; 3 Department of Preventive Medicine. Hospital Universitari de Bellvitge, Bellvitge Biomedical Research Institute (IDIBELL), L’Hospitalet de Llobregat, Barcelona, Spain; 4 Department of Medical Oncology, Catalan Institute of Oncology (ICO), L’Hospitalet de Llobregat, Barcelona, Spain; 5 Clinical Research in Solid Tumors Group, OncoBell Program, Bellvitge Biomedical Research Institute (IDIBELL), L’Hospitalet de Llobregat, Barcelona, Spain; 6 Unit of Human Anatomy, Department of Pathology and Experimental Therapeutics, Medical School, University of Barcelona, Barcelona, Spain

**Keywords:** Inflammation, Lung cancer, Survival, Lymphocyte-to-monocyte ratio, Neutrophil-to-lymphocyte ratio, Platelet-to-lymphocyte ratio

## Abstract

**Background:**

The aim of this study was to assess the effect of the lymphocyte-to-monocyte ratio (LMR), neutrophil-to-lymphocyte ratio and platelet-to-lymphocyte ratio on overall survival and disease-free survival in patients with lung cancer treated with radical surgery.

**Methods:**

We performed a retrospective review of patients with lung cancer who prospectively underwent radical resection between 2004 and 2012. Blood samples were taken as part of the preoperative workup. The inflammatory markers studied were absolute values of lymphocytes, monocytes, neutrophils and platelets, with subsequent calculation of ratios. Median follow-up was 52 months.

**Results:**

Two hundred and sixty-eight patients underwent surgery, of whom 218 (81.3%) were men. Mean age was 62.9 ± 8.7 years. A lymphocyte-to-monocyte ratio ≥ 2.5 was independently associated with longer disease-free survival (hazard ratio [HR] 0.476 (0.307–0.738), *p* = 0.001) and longer overall survival (HR, 0.546; 95% CI: 0.352–0.846; *p* = 0.007), in models adjusted for age, sex, stage, and type of resection. No other systemic inflammatory marker showed a significant association.

**Conclusion:**

Preoperative LMR is an independent prognostic factor of overall survival and recurrence-free survival in patients with surgically-resected early stage lung cancer.

## Background

Lung cancer causes the most cancer-related deaths worldwide. Increasing knowledge of tumour biology and multimodal treatments have helped improve treatment, and several prognostic factors exist, but overall survival remains poor, except in stage I, for which survival ranges between 60 and 80% [1, 2].

Prognostic factors related not to the tumour itself but to the patient’s general health status have been studied, including nutritional status and inflammatory status. Nutritional status is a prognostic factor in patients with lung cancer [3, 4], and our group has confirmed that nutritional status affects survival and postoperative outcomes in patients with lung cancer who undergo surgical resection [5].

Another prognostic factor that has been associated with survival and complications is inflammatory status. Multiple parameters can be used to determine the inflammatory status of a patient with cancer, but blood markers that are often used in preoperative assessment due to being simple to obtain are C reactive protein, absolute values of neutrophils, lymphocytes, monocytes and platelets, and the ratios of neutrophils to lymphocytes, platelets to lymphocytes and lymphocytes to monocytes. Certain levels of these parameters are associated with better or worse survival in various cancers, including lung cancer [6, 7].

The European Lung Cancer Working Group confirmed that patients with a high neutrophil count had worse survival [8]. Likewise, a high neutrophil-to-lymphocyte ratio and platelet-to-lymphocyte ratio are markers of worse prognosis in patients with cancer in general and particularly in lung cancer [9, 10]; however, within the inflammatory response, lymphocytes and monocytes both play an important role in postoperative outcomes following lung cancer resection [11, 12].

In this study, we assessed the effect of these systemic inflammatory markers on overall survival (OS) and disease-free survival (DFS) in patients with non-small cell lung cancer (NSCLC) who underwent radical resection with follow-up of at least 5 years.

## Methods

### Study population

**Fig. 1:**
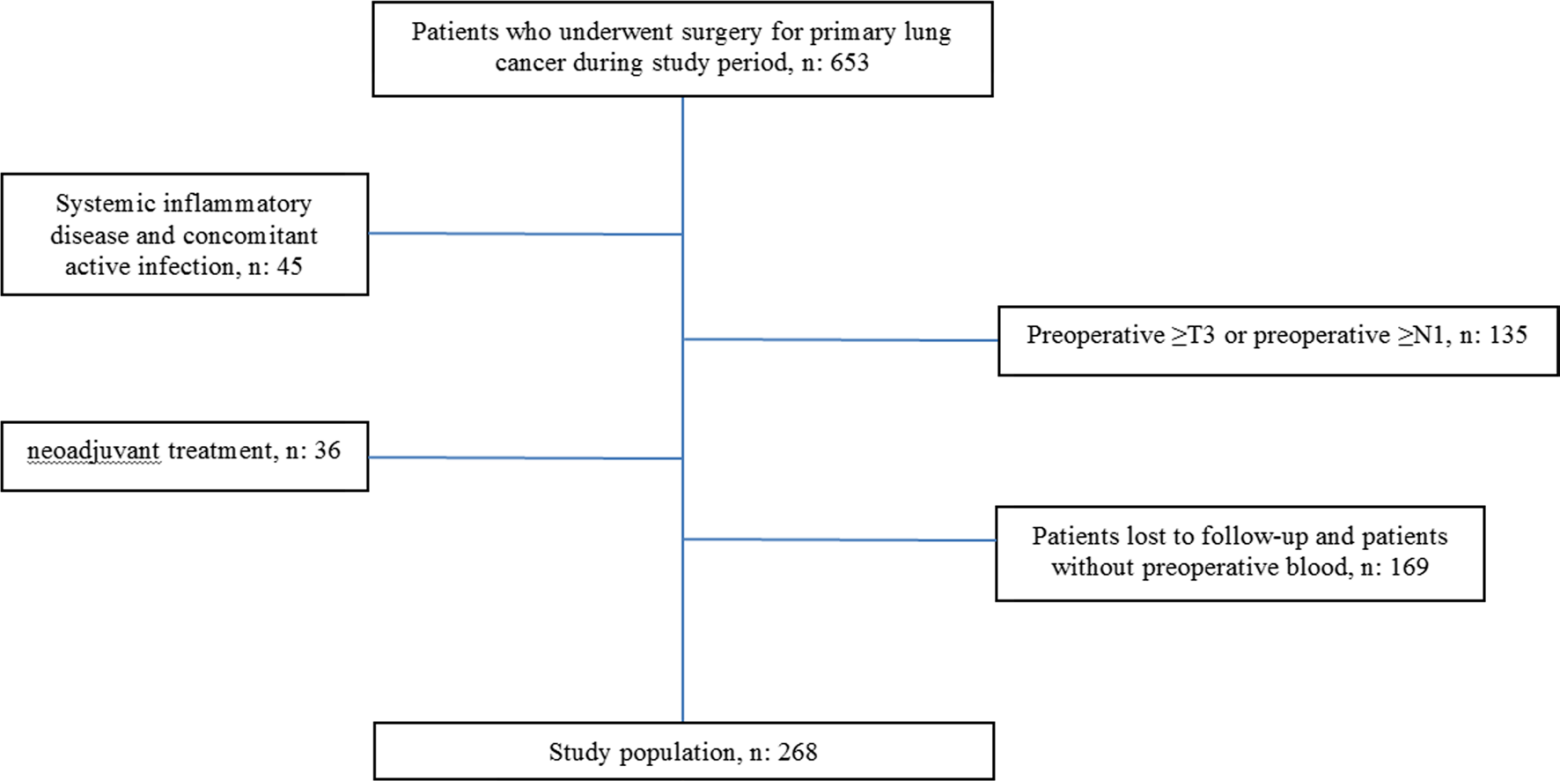
Flow chart of patient selection

We retrospectively reviewed a cohort of 653 patients who were treated with radical lung resection between January 2004 and December 2012. Patients with a history of systemic inflammatory disease, concomitant active infection, neoadjuvant treatment, preoperative stage ≥ T3, preoperative stage ≥ N1, patients lost to follow-up, or those for whom preoperative blood tests were not available were excluded (Fig. 1). Finally, we included 268 patients diagnosed with early clinical stage non-small cell lung cancer who underwent anatomic pulmonary resection with systematic lymph node dissection.

All patients underwent the same preoperative workup which included physical examination, full blood count and renal function tests, bronchoscopy, pulmonary function tests with diffusion studies, computed tomography (CT) and positron-emission tomography/computed tomography (PET-CT). All patients signed an informed consent form and the study was approved by the Institutional Review Board.

### Study variables

The variables studied were age, sex, comorbidities (smoking, diabetes mellitus, ischaemic heart disease, chronic obstructive pulmonary disease, and hypertension), type of surgery performed, pathological stage, tumour histology, disease-free survival and overall survival. The study aims did not include the association between these markers and postoperative complications.

The lymphocyte-to-monocyte ratio (LMR), neutrophil-to-lymphocyte ratio (NLR), and platelet-to-lymphocyte ratio (PLR) were calculated from the absolute neutrophil, platelet, monocyte and lymphocyte counts from routine preoperative testing performed 1–2 weeks before surgery at the same hospital.

### Follow-up

Routine follow-up was carried out at 1 month after surgery and included a full panel of blood tests and chest X-ray, then every 6 months with CT chest for the first 3 years, with annual checks thereafter, as per hospital protocol. If recurrence was suspected after surgery, the relevant tests, eg PET-CT, MRI brain, were requested according to the clinical scenario. Recurrences were classified as locoregional or distant metastases based on imaging. Locoregional recurrence was defined as recurrence in mediastinal or hilar lymph nodes or ipsilateral lung. Other recurrences were defined as distant metastases. All patients were seen by specialists and discussed by the multidisciplinary team to decide on treatment planning. Diagnosis of recurrence or distant metastases was evaluated and confirmed in the multidisciplinary unit.

### Statistical analysis

A descriptive analysis of the sample was performed as frequency and percentage for qualitative variables, and mean and standard deviation for quantitative variables if they followed a normal distribution (Kolmogorov–Smirnov, *p* value > 0.05), or median and interquartile range if not. Subsequently, Kaplan–Meier survival analysis was performed, and variables that were clinically relevant and statistically significant (Log rank *p* value < 0.05) and that did not show interaction between them were included in Cox regression. A receiver operating characteristics (ROC) analysis was performed to calculate the NLR, PLR, and LMR values that would have the greatest sensitivity and specificity. The statistical software SPSS v 16.0 was used.

## Results

Two hundred and sixty-eight patients were included in the study, of whom 218 (81.3%) were men. The mean age was 62.9 ± 8.7 years. Clinical, surgical, and pathological features and blood values of the study population are shown in Table 1. Twenty-five patients (9.2%) received tumour-specific adjuvant treatment. The median follow-up time was 52 months. One hundred patients (37.3%) developed recurrence, of whom 74 (27.6%) had distant metastases and 26 (9.7%) had locoregional recurrence. Twelve patients (4.5%) developed second primary lung tumours and underwent new radical surgery. One hundred and fifty-nine (57.3%) patients were still alive at the end of follow-up; 109 (42.7%) patients died.

**Table 1:** Clinical, surgical, pathological and inflammatory parameters for the study population

	N (%) or mean ± SD or median (range)
Males	268 (81.3%)
Age (years)	62.9 ± 8.7
Smoker	205 (76.5%)
Diabetes mellitus	57 (21.3%)
Ischaemic cardiomyopathy	33 (12.3%)
COPD	100 (37.3%)
Hypertension	103 (38.4%)
Dyslipidaemia	99 (36.9%)
Surgical procedure	
Lobectomy	224 (83.6%)
Pneumonectomy	14 (5.2%)
Sub-lobar resection	38 (11.2%)
Pathologic stage	
Ia/Ib	173 (64.5%)
IIa/IIb	74 (27.6%)
IIIa/IIIb	19 (7.1%)
IV	2 (0.8%)
Histological type	
Adenocarcinoma	168 (62.7%)
Squamous cell carcinoma	79 (29.5%)
Large cell carcinoma	18 (6.7%)
Other	3 (1.1%)
Neutrophils, × 10^9^/L	4.47 (1.63)
Lymphocytes, × 10^9^/L	2.14 (1.59)
Monocytes, × 10^9^/L	0.57 (0.20)
Platelets, × 10^9^/L	243 (79.71)
LMR	3.97 (1.97)
NLR	2.38 (1.23)
PLR	131.59 (65.03)

*COPD* chronic obstructive pulmonary disease, *LMR* lymphocyte-to-monocyte ratio, *NLR* neutrophil-to-lymphocyte ratio, *PLR* platelet-to-lymphocyte ratio

**Fig. 2:**
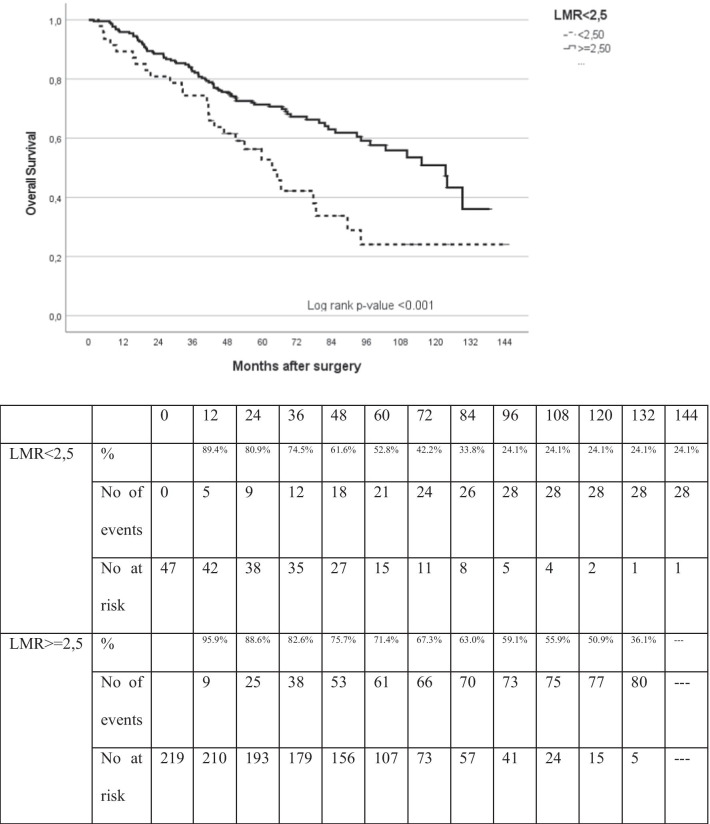
Kaplan–Meier analysis of overall survival (OS) based on LMR

**Fig. 3:**
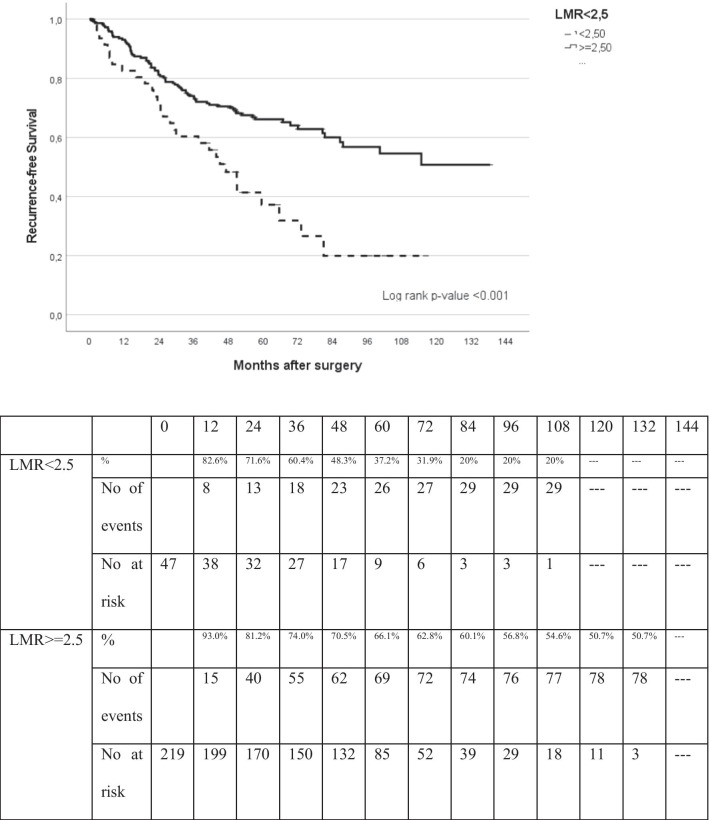
Kaplan–Meier analysis of disease-free survival (DFS) based on LMR

Following ROC analysis, the values with the greatest sensitivity and specificity were 2.5 for NLR and LMR and 150 for PLR. Disease-free survival and OS were evaluated after splitting patients according to these proposed maker levels (Table 2). An LMR ≥ 2.5 was a clear prognostic factor for higher overall survival and lower recurrence in patients with surgically-resected lung cancer (*p* = 0.001) (Figs. 2, 3). On univariate analysis, age, pathological stage and LMR ≥ 2.5 were significantly associated with higher disease-free survival (HR, 0.444; 95% CI 0.289–0.683; *p* = 0.001). These variables remained statistically significant on multivariate analysis (HR, 0.476; 95% CI 0.307–0.738; *p* = 0.001; Table 3) after adjusting for age, sex, pathological stage and histology.

**Table 2:** Distribution of patients according to LMR

Study population, n: 268	LMR < 2.5	LMR ≥ 2.5
No. of patients	47	221
No. of patients who died during the follow-up period	28	81
No. of patients who died or had recurrence during the follow-up period	35	107

*LMR* lymphocyte-to-monocyte ratio

**Table 3:** Univariate and multivariate analysis of disease-free-survival (DFS)

Covariables	Hazard ratio	95% CI	*p* value
*Univariate analysis*			
PLR ≥ 150	0.986	0.654–1.488	0.948
NLR ≥ 2.5	1.203	0.821–1.761	0.343
LMR ≥ 2.5	0.444	0.289–0.683	0.001
Age	1.018	0.996–1.040	0.112
Sex (female)	0.970	0.595–1.579	0.901
Pathological stage			0.051*
I	1.000		
II	1.686	1.126–2.526	0.011
III	1.793	0.812–3.958	0.148
IV	2.536	0.350–18.380	0.357
Histological type			0.219*
Adenocarcinoma	1.000		
Squamous cell carcinoma	0.618	0.384–0.995	0.048
Large cell carcinoma	0.792	0.381–1.646	0.532
Other	0.568	0.138–2.343	0.434
*Multivariate analysis*			
LMR ≥ 2.5	0.476	0.307–0.738	0.001
Age	1.029	1.005–1.054	0.017
Sex			
Male	1.000		
Female	1.171	0.693–1.977	0.556
Pathological stage			0.027*
I	1.000		
II	1.779	1.174–2.695	0.007
III	1.878	0.848–4.162	0.120
IV	3.101	0.406–23.695	0.275
Histological type			0.053*
Adenocarcinoma	1.000		
Squamous cell carcinoma	0.527	0.322–0.862	0.011
Large cell carcinoma	0.619	0.294–1.301	0.205
Other	0.541	0.127–2.306	0.407

*LMR* lymphocyte-to-monocyte ratio, *NLR* neutrophil-to-lymphocyte ratio, *PLR* platelet-to-lymphocyte ratio

^*^Linear trend *p* value

Regarding overall survival, univariate analysis showed that age, pathological stage and LMR ≥ 2.5 were associated with better overall survival (HR, 0.488; 95% CI 0.317–0.751; *p* = 0.001). These variables were also significant on multivariate analysis (HR, 0.546; 95% CI 0.352–0.846; *p* = 0.007; Table 4) after adjusting for age, sex, pathological stage and histology. NLR and PLR were not found to be prognostic factors for overall survival or disease-free survival.

**Table 4:** Univariate and multivariate analysis of overall survival

Covariables	Hazard ratio	95% CI	*p* value
*Univariate analysis*			
PLR ≥ 150	1.151	0.772–1.715	0.494
NLR ≥ 2.5	1.175	0.806–1.714	0.401
LMR ≥ 2.5	0.488	0.317–0.751	0.001
Age	1.037	1.013–1.060	0.002
Sex (female)	0.831	0.481–1.436	0.507
Pathological stage			0.026*
Stage I	1.000		
Stage II	1.718	1.148–2.571	0.009
Stage III	1.980	0.975–4.017	0.059
Stage IV	2.296	0.317–16.631	0.411
Histological type			0.999*
Adenocarcinoma	1.000		
Squamous cell carcinoma	0.991	0.651–1.509	0.967
Large cell carcinoma	1.041	0.518–2.091	0.909
Other	0.986	0.135–7.190	0.989
*Multivariate analysis*			
LMR ≥ 2.5	0.546	0.352–0.846	0.007
Age	1.041	1.017–1.066	0.001
Sex			
Male	1.000		0.966
Female	1.013	0.564–1.819	
Pathological stage			0.013*
I	1.000		
II	1.783	1.179–2.697	0.006
III	2.144	1.047–4.389	0.037
IV	4.083	0.521–31.995	0.180
Histological type			0.830*
Adenocarcinoma	1.000		
Squamous cell carcinoma	0.812	0.525–1.255	0.348
Large cell carcinoma	0.923	0.457–1.864	0.824
Other	0.929	0.122–7.056	0.943

*LMR* lymphocyte-to-monocyte ratio, *NLR* neutrophil-to-lymphocyte ratio, *PLR* platelet-to-lymphocyte ratio

^*^Linear trend *p* value

## Discussion

The association between inflammation and cancer was first described years ago and has been the subject of much study. O’Callaghan et al. [13] described the role of inflammation in the etiopathogenesis of lung cancer, and multiple studies have confirmed the prognostic value of inflammation in lung cancer outcomes, for both local and advanced disease [10, 14].

Our study included 268 patients who underwent resection and prospective follow-up for at least 5 years, and demonstrates that an LMR ≥ 2.5 is an independent positive prognostic factor for disease-free survival and overall survival. Although this ratio has not been studied extensively in cancer and particularly bronchogenic cancer, our findings are in line with those obtained by other groups. Xia et al. [15], in 439 patients with stage I NSCLC, demonstrated a positive association between LMR and overall survival and a greater risk of distal metastases with lower LMR. However, Asian populations may behave differently from European populations in terms of blood markers, so their results will need to be validated in a Western population.

In 2018, Chen et al. [16] published a series of 577 surgical patients in stage IB NSCLC who had undergone pneumonectomy. They found that LMR and PLR were independent prognostic factors for OS. In our series, LMR was an independent prognostic factor for OS and DFS. However, we did not find a statistically significant association for PLR as a prognostic factor. One explanation could be that the patients requiring pneumonectomy are usually patients with larger tumours or with greater intrapulmonary lymph node involvement, so they are likely to have higher baseline levels of inflammation. In our study only 5.2% of the patients had undergone pneumonectomy, while 100% of those in the study by Chen et al. had undergone pneumonectomy, with pleural invasion in 39% of the cases.

The immunological basis for our findings is that lymphoid cells play a primordial role in the control, proliferation, and migration of tumour cells [17]. In cervical cancer, it has been observed that lymphocytes act as essential components of the immune response, and low levels of lymphocytes in the peripheral blood and tumour stroma lead to a weaker immune response against the cancer cell [18]. In contrast, the presence of high levels of monocytes and their derivatives induces immunosuppression and tumour neoangiogenesis. In addition, intratumour macrophages, derived directly from tissue monocytes, facilitate tumour cell migration by secreting mediators that degrade the extracellular matrix and attract more intratumour monocytes/macrophages, leading to greater tumour aggressiveness both locally and distally [19–22].

In nonsurgical treatments such as stereotactic radiation therapy, several groups have described the effect of NLR and PLR in terms of local recurrence. Canon et al. [10] found that PLR should be used as a prognostic factor for DFS. In our study, we did not find such an association, but we must bear in mind that the inflammatory status in the nonsurgical population is probably different: although in some cases patients decline surgery, in many cases they are unsuitable for surgery due to comorbidities.

The interaction between nutritional status, systemic inflammatory status and tumour inflammatory status plays a key role in postoperative outcomes and prognosis in patients with lung cancer [23]. This explains why a higher or lower LMR confers better or worse disease prognosis among patients undergoing surgery, who theoretically will have better long-term outcomes.

The present study has the limitations of being a single-centre retrospective study. Also, although the preoperative testing used to obtain the ratios was the same for all patients, the findings have not been validated in an independent cohort. The ideal cut-off point for the ratio is difficult to establish, but according to our results, the value with the greatest sensitivity and specificity was 2.5; values above or below this showed significant differences in overall survival and disease-free survival, in line with other published studies [15–17, 24, 25].

The number of patients included, more than 200, the consistency of the blood testing method, the follow-up time and the prospective format of data recording make the data presented relevant for future research.

## Conclusions

The findings from this study in a cohort of patients who underwent surgery for NSCLC confirm that the lymphocyte-to-monocyte ratio is a convenient preoperative biomarker that could provide valuable information on the probability of recurrence and overall survival in this population.

## Data Availability

The datasets used and/or analysed during the current study are available from the corresponding author on reasonable request.

## Abbreviations

LMR: Lymphocyte-to-monocyte ratio
NLR: Neutrophil-to-lymphocyte ratio
PLR: Platelet-to-lymphocyte ratio
OS: Overall survival
DFS: Disease-free survival
HR: Hazard ratio
NSCLC: Non-small cell lung cancer
CT: Computed tomography
PET-CT: Positron-emission tomography/computed tomography
ROC: Receiver operating characteristics
